# ﻿*Flospes* gen. nov. (Orthoptera, Trigonidiidae, Trigonidiinae), a genus of swordtail crickets from China, with two new species and new combinations

**DOI:** 10.3897/zookeys.1090.77830

**Published:** 2022-03-24

**Authors:** Zhi-Xin He, Li-Bin Ma, Tao Zhang, Xiao-Lan Miao

**Affiliations:** 1 College of Life Sciences, Shaanxi Normal University, Xi’an, 710119, China

**Keywords:** Grylloidea, new genus, silent crickets, taxonomy, Trigonidiini

## Abstract

We propose the genus *Flospes***gen. nov.** for two new species, *Flospesguangxiensis***sp. nov.** and *Flospesviridipennis***sp. nov.**, obtained from Guangxi and Hainan provinces, China, respectively, based on male genitalia traits. Three other species with similar genitalia are placed in the new genus: *Flospesfujianensis* (Wang et al., 1999), **comb. nov.**, *Flospeshainanensis* (He et al., 2010), **comb. nov.**, and *Flospesdenticulatus* (Liu & Shi, 2011), **comb. nov.** The new species are described, illustrated, and their ranges are given.

## ﻿Introduction

There are 49 genera and 658 species in the Trigonidiinae (Orthoptera, Trigonidiidae) (Cigliano et al. 2021). In recent years, male genitalia have been used to identify crickets. It used to be relatively uncommon, but people loved to utilize acoustical structures to identify Trigonidiinae species (e.g., if the tegmen had a mirror or stridulatory vein and whether the tympanum was present) (Chopard 1936, 1951, 1969; Otte and Alexander 1983). Otte (2006) even speculated that classifying them by genital characteristics would result in a jumble of taxa. In our research, however, we discovered considerable intraspecific differences in these traits. Genitalia morphology is a crucial characteristic for delimiting genera of Grylloidea, just as it is for other taxa (Gorochov 2015; Gorochov et al. 2018). As a result, we handle trigonidiine taxa by concentrating on male genitalia and propose a new genus for two new species (*Flospes* gen. nov., *Flospesguangxiensis* sp. nov., and *Flospesviridipennis* sp. nov.).

Three more species should also be added to the new genus. According to the same kind of male genitalia when the genus was formed, *Amusurgushainanensis* (= *Sectushainanensis*) He et al., 2010 was included in *Sectus*Ma and Pan (2019). Due of its similar appearance to *S.hainanensis*, He et al. (2020) placed *Amusurgusfujianensis* (= *Sectusfujianensis*) (Wang et al. 1999) in the same genus. However, both *S.hainanensis* and *S.fujianensis* were found to be comparable to the new genus in terms of male genitalia or appearance in this study. For example, the epiphallus of *S.fujianensis* has lateral lobes, as in the new genus, while the type species of *Sectus* does not. *Metiochodesdenticulatus* Liu & Shi, 2011 was formerly classified as a species of the genus *Metiochodes* Chopard, 1932, but its male genitalia matches that of the new genus. As a result, these species are here classified in *Flospes* gen. nov.: *Flospeshainanensis* comb. nov., *Flospesfujianensis* comb. nov., and *Flospesdenticulatus* comb. nov.

## ﻿Materials and methods

Specimens were firstly preserved in ethanol during fieldwork and then pinned and dry to be maintained in the laboratory. Photographs of specimens were obtained using a VHX-6000 Super-high magnification lens zoom 3D microscope (Keyence, Osaka, Japan). We dissected male genitalia from softened specimens and cleaned using aqueous protease, and made photomicrographs of genitalia using ToupCam Digital camera and bundled software (ToupTek, Hangzhou, China). Terminology used to describe the male genitalia follows Desutter-Grandcolas (1987). The specimens are deposited at the Museum of Flora and Fauna of Shaanxi Normal University, Xi’an, China (**SNNU**).

### ﻿Measurements

All specimens were measured using a ToupCam Digital camera and bundled software (ToupTek, Hangzhou, China). All the measurements are in millimeters (mm).

### ﻿Abbreviations

**BL** body length (from head to apical hindwing);

**PL** pronotal length;

**TL** tegmen length;

**HFL** hind femur length,

**OL** ovipositor length;

**ep lb** epiphallic lateral lobe;

**ecp** ectoparamere;

**ecp ub** upper branch of ectoparamere;

**ecp lb** lower branch of ectoparamere;

**r** rami;

**ec ap** ectophallic apodeme;

**en ap** endophallic apodeme.

## ﻿Taxonomy

**Subfamily Trigonidiinae Saussure, 1874**

### Tribe Trigonidiini Saussure, 1874

#### ﻿Genus Flospes

Taxon classificationAnimaliaOrthopteraTrigonidiidae

Ma & He gen. nov.

11872F6B-4EB5-5C0B-B5B0-4476E97F52ED

http://zoobank.org/33ADF11D-F7A6-4886-8061-42AB035ED6A6

##### Type species.

*Amusurgusfujianensis* (=*Flospesfujianensis*).

##### Etymology.

The genus name “Flospes” is a Latin word (= flower), which refers to the colorful body of the members of the genus (the fore and median femora are proximally black and distally white, the hind femur bears a dark brown band, and the cercus is black and white).

##### Diagnosis.

Head almost as wide as anterior margin of pronotum. Frons slightly convex. Maxillary palpi black and white. Tegmen similar in both sexes (male lack of stridulatory apparatus). The internal tympanum large and long-oval, and the external one replaced by a small pit. The hind tibia bears three pairs of dorsal spurs. The legs and cercus black and white. The lateral lobes of epiphallus rod-like and ectoparamere enormously enlarged (much wider than epiphallic lateral lobe). The apex of female ovipositor expanded, blade-like and reddish brown.

##### Remarks.

Similar to *Amusurgus*, the members of them are silent, pubescent and bearing rod-like epiphallic lateral lobes, but the species of the new has colorful legs and cercus, as well as ectoparamere that is enormously enlarged and almost membranous. The new genus is distinguished from *Sectus* by the absence of stridulatory apparatus and the presence epiphallic lobes. It differs from the genus *Metiochodes* Chopard, 1932 in that its ectoparamere is enlarged and membranous.

#### ﻿Key to known species of *Flospes* (male adults)

**Table:** 

1	Veins green (Fig. 9)	***Flospesviridipennis* sp. nov.**
–	Veins yellow (Fig. 5)	**2**
2	Epiphallus very short, almost without lateral lobe	***Flospeshainanensis* comb. nov.**
–	Epiphallus normal, bearing conspicuous lateral lobes and dorsally viewed as following (Figs 8, 12)	**3**
3	Epiphallic lateral lobe apically acute	***Flospesdenticulatus* comb. nov.**
–	Epiphallic lateral lobe apically blunt	**4**
4	Ectoparamere apically rounded	***Flospesfujianensis* comb. nov.**
–	Ectoparamere apically rectangular	***Flospesguangxiensis* sp. nov.**

#### ﻿ Flospes denticulatus comb. nov.

Taxon classificationAnimaliaOrthopteraTrigonidiidae

43468707-4AAC-57EE-97BC-341E5DAF5C6D

Metiochodes denticulatus Liu & Shi, 2011: 2

##### Holotype information.

Type locality: China. Guizhou, Rongjiang, Xiaodanjiang. Deposited at Hebei University Museum (HBUM), Hebei, China (not examined).

##### Distribution

**Figure 1.**
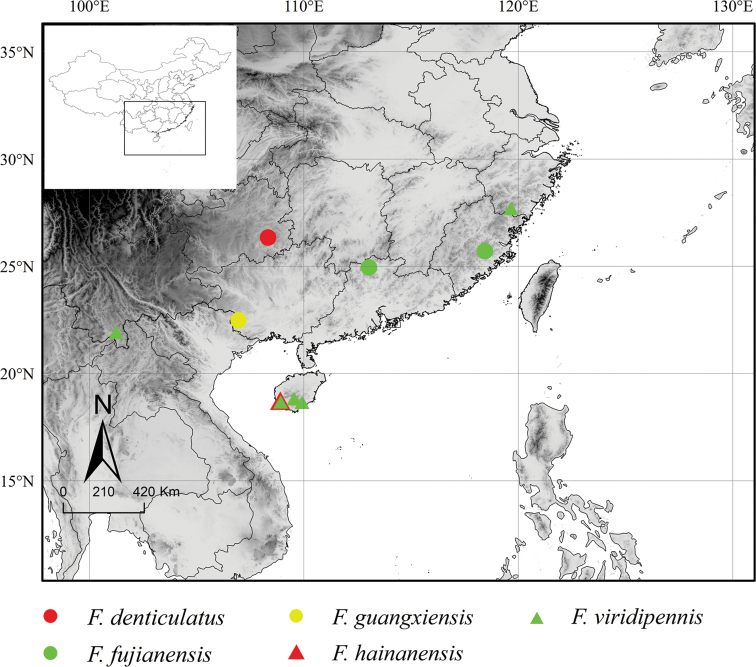
Distribution of *Flospes* species in China.

(Fig. 1). China (Guizhou).

##### Remarks.

This species was initially arranged in the genus *Metiochodes*, and its features of appearance and male genitalia are consistent with the characteristics of the new genus.

#### ﻿ Flospes fujianensis comb. nov.

Taxon classificationAnimaliaOrthopteraTrigonidiidae

CB190F40-C9CD-504A-B776-48566143328E

**Figure 2.**
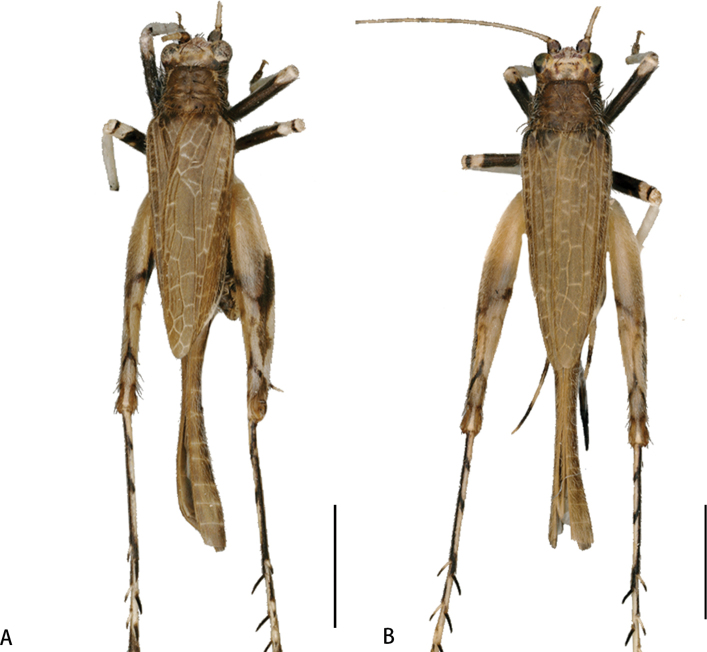
Habitus photographs of *Flospesfujianensis***A** male **B** female. Scale bar: 2 mm

**Figure 3.**
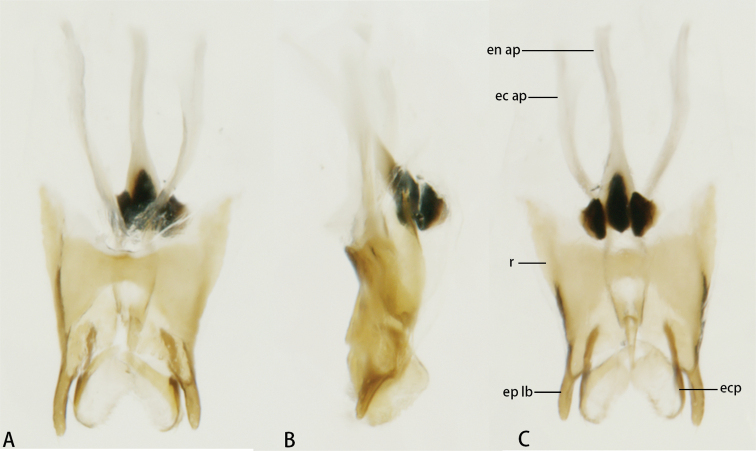
Male genitalia of *Flospesfujianensis***A** dorsal view **B** lateral view **C** ventral view.

**Figure 4.**
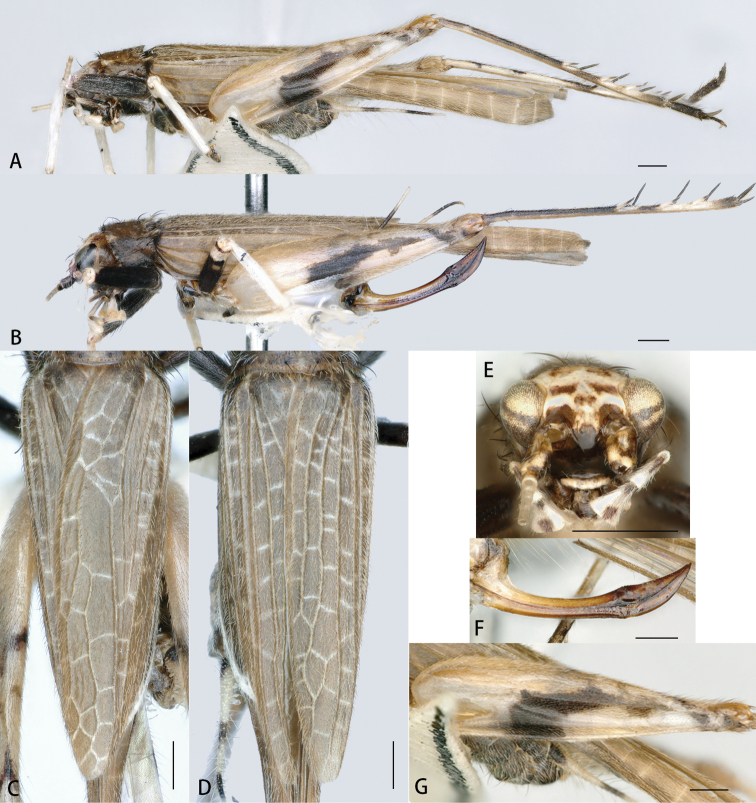
*Flospesfujianensis* comb. nov. **A** lateral view of male **B** lateral view of female **C** male tegmen **D** female tegmen **E** face in anterior view **F** female ovipositor in lateral view **G** lateral view of hind femur. Scale bars: 0.5mm.

Chinese name: 福建花蛉蟋 Figs 1 , 2 , 3 , 4

Amusurgus fujianensis Wang, Zheng & Wu, 1999: 114
Amusurgus (Paranaxipha) fujianensis (= Sectusfujianensis), He et al. 2010: 60; He et al. 2020:126. misidentification of F.viridipennis sp. nov.

##### Materials examined.

**China**: 3 males, 3 females; Guangdong, Nanling National Nature Reserve, bush leaves, 24.93°N, 113.04°E, 5.VIII.2019, Zhixin He & Tao Zhang, sweep net, leg. (SNNU).

##### Redescription.

**Male** (Figs 2A, 4A, C, E, G). Body size small, pubescent. Head small, slightly broader than anterior margin of pronotum. Frons slightly narrower than antennal scape. Eyes large and strongly protruding to sides. Clypeus narrow, upper margin straight, and lower margin medially convex. Labrum shield-like, medially plump, and apically concave. Three apical joints of maxillary palpi distinctly elongate, and fifth joint apically truncated. Pronotum transverse, anterior margin straight, and posterior margin slightly and medially convex. Tegmina pubescent. Six primary veins staggered with numerous transverse veins between them. Visible part of hindwing is half length of tegmen. Internal tympanum large and long-oval, and external one replaced by a small pit. Hind tibia bearing three pairs of dorsal spurs.

***Genitalia*** (Fig. 3A–C). Lateral lobes of epiphallus stick-like, apically blunt, and almost straight in dorsal view. Ectoparamere spoon-like, with sclerotized margin and membranous remainder portion. Rami very short. Ectophallic and endophallic apodemes greatly surpass the rami.

**Female** (Figs 2B, 4B, D, F). Resembles male. Longitudinal veins of tegmen parallel, with a few pale transverse veins forming several rectangular cells (filled with brown). Ovipositor not surpassing hindwings. Dorsal and ventral margins of basal ovipositor smooth and faintly narrowing, and both margins of apical part slightly denticulated and apically bent upwards.

**Coloration.** Body yellowish-brown. Pronotum brown. Fore and middle femur darkly colored. Hind femur bearing two dark bands (middle one and apical one). Tarsus darkly color. Cercus black and white.

**Measurements.** Male: BL 5.08–5.73, PL 0.66–0.75, TL 4.00–4.60, HFL 4.22–4.69. Female: BL 5.61–6.42, PL 0.90–1.04, TL 4.28–4.77, HFL 4.59–5.11, OL 2.40–2.74.

##### Distribution

**(Fig. 1).** China (Fujian, Guangdong).

##### Remarks.

This species was first described from Fujian Province, China. Its primary characteristics are body brown, leg and cercus black and white, tegmen brown, and armed with rod-like lateral lobe on the epiphallus and an enormously enlarged, rounded ectoparamere. He et al. (2010) described some specimens found in Hainan, Zhejiang, and Yunnan provinces as *A.fujianensis*, but these specimens bear a greenish-brown tegmen and squared ectoparamere. We checked specimens collected from Hainan Province and discovered that they are consistent with the description of “*A.fujianensis*” in He et al. (2010). Our specimens from Guangdong Province are consistent with the original description of *A.fujianensis* by Wang et al. (1999). As a result, we redescribe *A.fujianensis* here from our Guangdong specimens and judge that “*A.fujianensis*” of He et al. (2010) is a misidentification (see Remarks below under *Flospesviridipennis* He & Ma, sp. nov.).

#### ﻿ Flospes guangxiensis

Taxon classificationAnimaliaOrthopteraTrigonidiidae

He & Ma sp. nov.

ED8B9894-64C4-508A-8639-0605A867688D

http://zoobank.org/185101ED-7820-41C2-84BA-B22DA49EE4E6

**Figure 5.**
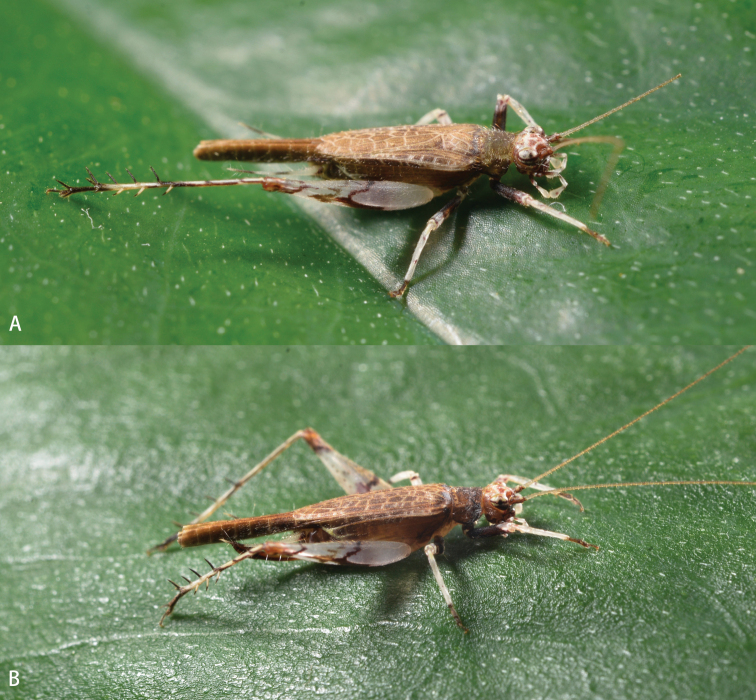
Habitus (alive) of *Flospesguangxiensis* sp. nov. on a leaf **A** male **B** female.

**Figure 6.**
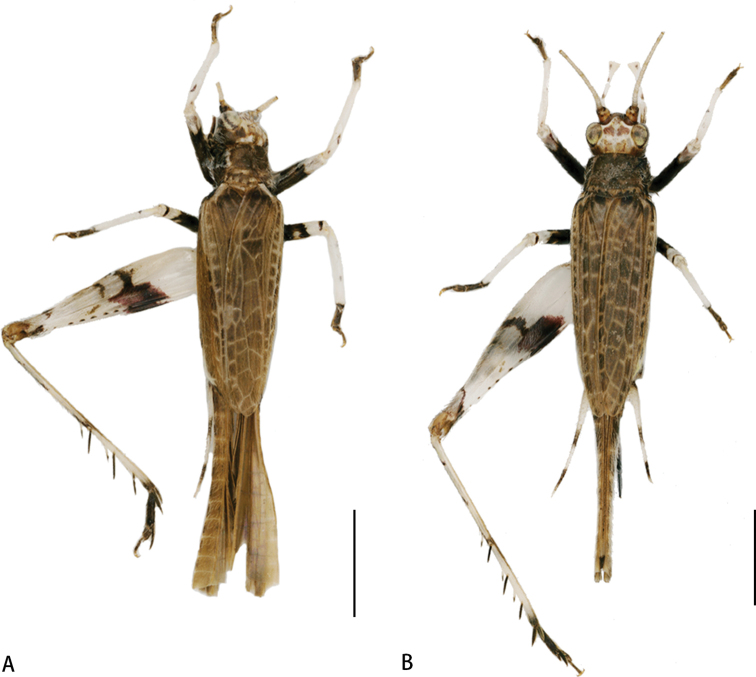
Habitus photographs of *Flospesguangxiensis* sp. nov. **A** male **B** female. Scale bar: 2 mm.

**Figure 7.**
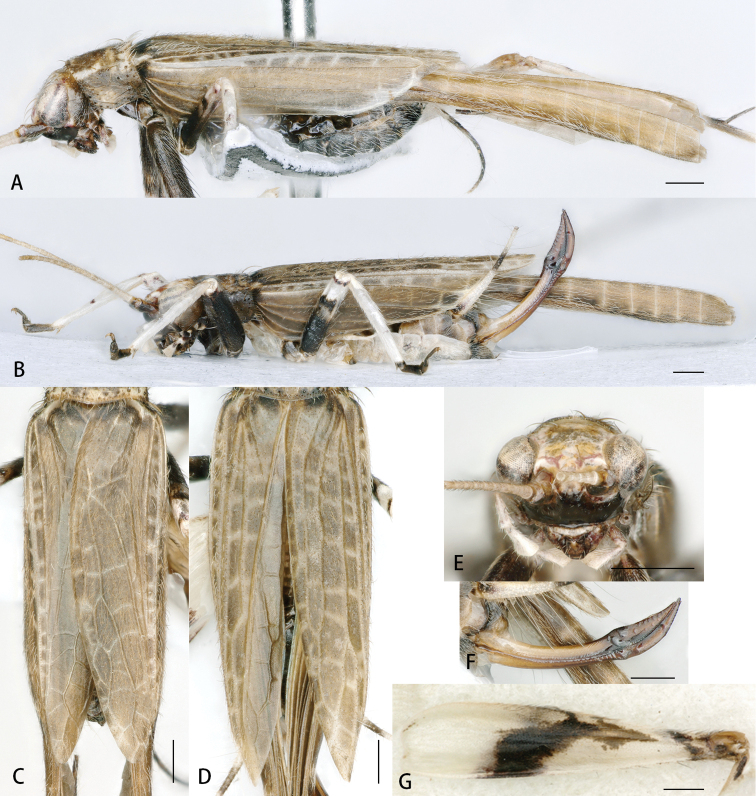
*Flospesguangxiensis* sp. nov. **A** lateral view of male **B** lateral view of female **C** male tegmen **D** female tegmen **E** face in anterior view **F** female ovipositor in lateral view **G** lateral view of hind femur. Scale bars: 0.5mm.

**Figure 8.**
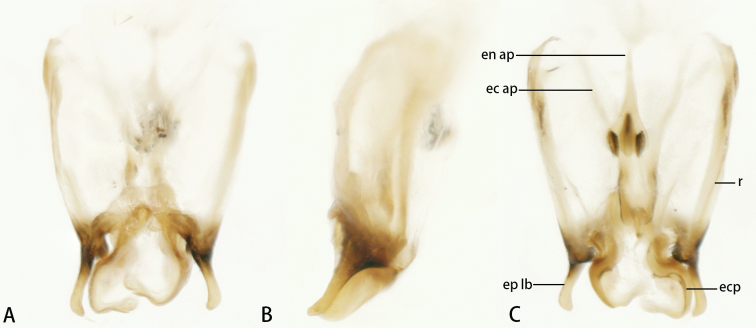
Male genitalia of *Flospesguangxiensis* sp. nov. **A** dorsal view **B** lateral view **C** ventral view.

Chinese name: 广西花蛉蟋 Figs 1 , 5 , 6 , 7 , 8

##### Type materials.

***Holotype*. China**: Male, Guangxi, Longzhou, Nonggang National Nature Reserve, bush leaves, 2.X.2021, 22.46°N, 106.96°E, Zhixin He & Ning Wang, sweep net, leg. ***Paratypes*.** 1 male, 2 females, same data as holotype (SNNU).

##### Description.

**Male** (Figs 5A, 6A, 7A, C, E, G). Body size small. Head small, slightly broader than anterior of pronotum. Frons as wide as antennal scape. Eyes large and protruding laterally. Three apical joints of maxillary palpi distinctly elongate, and the fifth joint apically truncated. Pronotum transverse, posterior margin broader than the anterior one. Tegmen extending over abdominal apex, and bearing six staggered primary veins with many transverse veins between them. The visible part of hindwing half length of tegmen. Internal tympanum large and long-oval, and external one shaped like a small pit. The hind tibia bears three dorsal spurs on each side.

***Genitalia*** (Fig. 8A–C). Lateral lobes of epiphallus stick-like, apically blunt, and inward curved in dorsal view. Ectoparamere roughly rectangular with the marginal part sclerotized and the remainders membranous. Ectophallic and endophallic apodemes short and not surpass rami.

**Female** (Figs 5B, 6B, 7B, D, F). Resembles male but slightly smaller. Longitudinal veins of tegmen parallel and producing rectangular cells (fill with dark brown) with a few pale transverse veins. Ovipositor not surpassing hindwings. Dorsal and ventral margins of the basal of ovipositor smooth and faintly narrowing, and both margins of apical part slightly denticulated and apically curved upwards.

**Coloration.** Body yellowish-brown. Pronotum dark brown. Most of the fore and middle femur darkly colored, but with apex pale. Hind femur medially bears a dark brown band. Tarsus darkly colored. Cercus black and white.

**Measurements.** Male: BL 5.68–5.93, PL 0.82–0.91, TL 4.22–4.64, HFL 3.82–4.21. Female: BL 5.87–6.21, PL 0.76–0.82, TL 4.21–4.43, HFL 4.07–4.33, OL 2.25–2.51.

##### Etymology.

The name refers to the province of China where the type locality is located.

##### Distribution

(Fig. 1). China (Guangxi).

##### Remarks.

This species is similar in appearance to *F.fujianensis* and *F.denutilatus* but differs in its squared ectoparamere (that of *F.fujianensis* is round) and blunt apex of epiphallic lateral lobe (that of *F.denutilatus* is acute). The transverse cells of female tegmen of the new are more darker than that of *F.fujianensis*.

#### ﻿ Flospes hainanensis comb. nov.

Taxon classificationAnimaliaOrthopteraTrigonidiidae

D49D6C5B-5DD1-5A1B-96CF-0F9E23668F8C

Amusurgus (Paranaxipha) hainanensis He et al., 2010: 60
Sectus hainanensis , Ma & Pan, 2019: 567

##### Holotype information.

Type locality: China. Hainan, Ledong, Jianfeng. Deposited at East China Normal University, Biology of History Museum (HSUN), Shanghai, China (not examined).

##### Distribution

(Fig. 1). China (Hainan).

##### Remarks.

This species has very unique male genitalia; the epiphallus is relatively short and the lateral lobe is nearly absent. These features correspond to some other genera of Trigonidiinae (e.g., *Sectus* and *Anaxiphomorpha*), and even species of Nemobiinae. This species has been assigned in the genus *Sectus* according to the genitalic form (Ma and Pan 2019). Because the type species of *Sectus* bears acoustical devices (tympana and stridulatory vein and mirror, etc.) and can sing, the placement of *F.hainanensis*, a silent cricket species, in *Sectus* is unsuitable. The similar appearance of this species with *F.fujianensis* makes it more suitable to move it to the new genus.

#### ﻿ Flospes viridipennis

Taxon classificationAnimaliaOrthopteraTrigonidiidae

He, Ma & Zhang sp. nov.

2C47CA05-C918-52FC-B9BF-DEC780240944

http://zoobank.org/5B3364B1-B998-4433-8DBD-27A324D288D5

**Figure 9.**
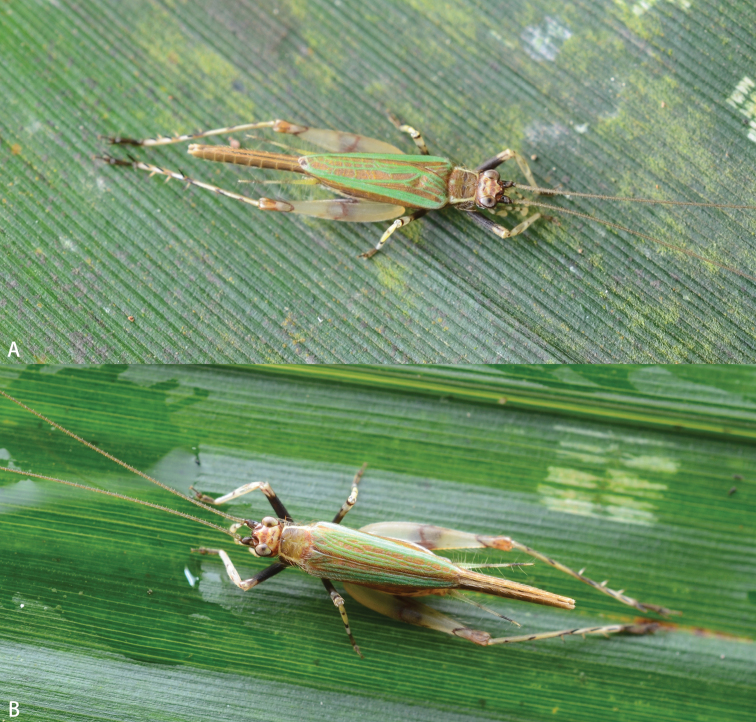
Habitus (alive) of *Flospesviridipennis* sp. nov. on leaf **A** male **B** female.

**Figure 10.**
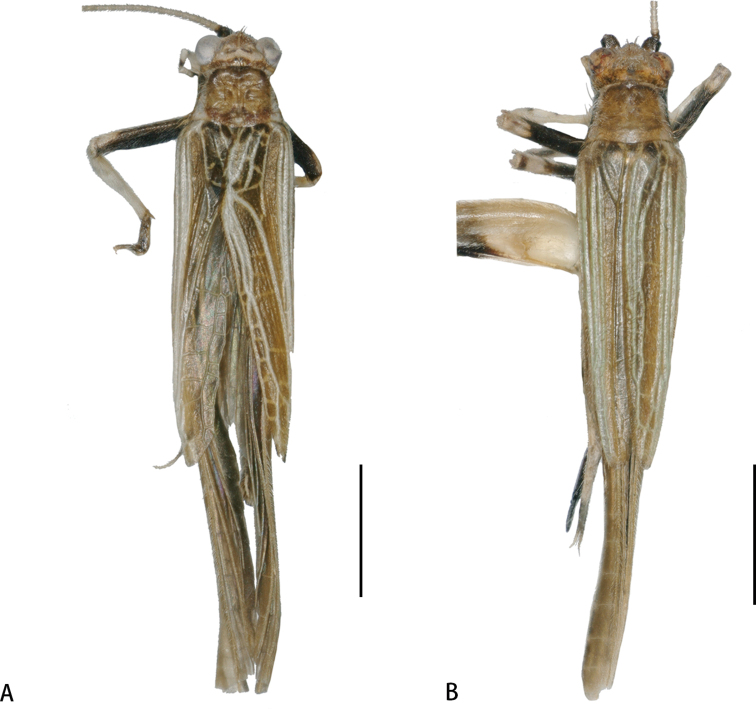
Habitus photographs of *Flospesviridipennis* sp. nov. **A** male **B** female. Scale bars: 2 mm.

**Figure 11.**
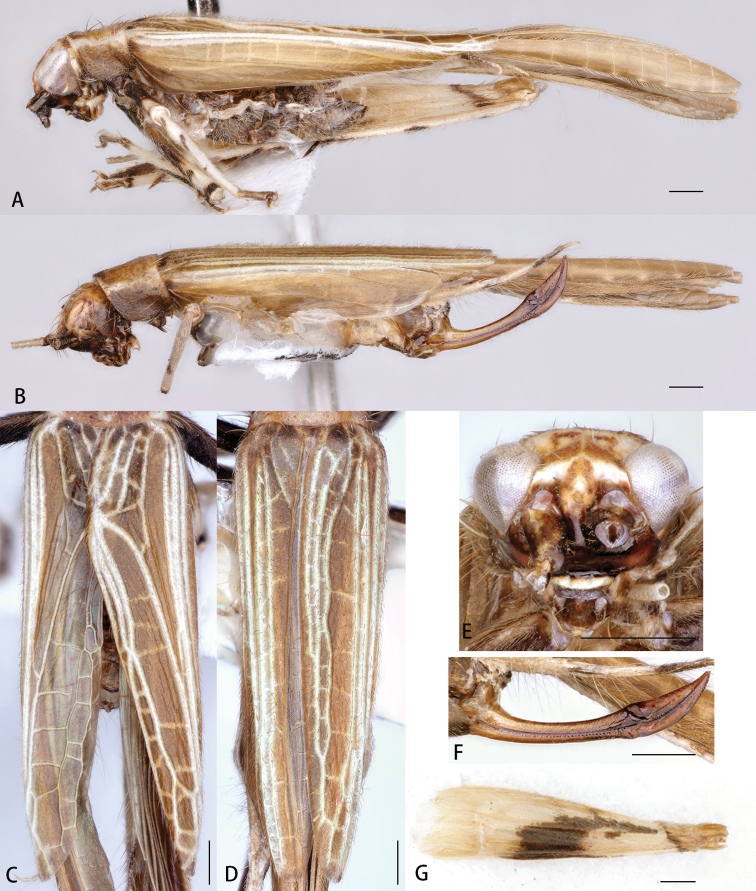
*Flospesviridipennis* sp. nov. **A** lateral view of male **B** lateral view of female **C** male tegmen **D** female tegmen **E** face in anterior view **F** female ovipositor in lateral view **G** lateral view of hind femur Scale bars: 0.5mm.

**Figure 12.**
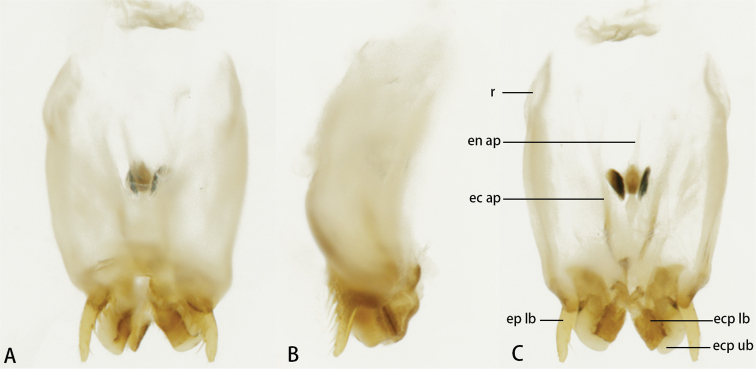
Male genitalia of *Flospesviridipennis* sp. nov. **A** dorsal view **B** lateral view **C** ventral view.

Chinese name: 青翼花蛉蟋 Figs 1 , 9 , 10 , 11 , 12

Amusurgus (Paranaxipha) fujianensis (= Sectusfujianensis), He et al. 2010: 60; He et al. 2020: 126. misidentification of F.viridipennis sp. nov.

##### Type materials.

***Holotype*. China**: Male, Hainan, Lingshui, Diaoluoshan National Nature Reserve, bush leaves, 14.IX.2019, 18.66°N, 109.92°E, Zhixin He & Tao Zhang, sweep net, leg. ***Paratypes*.** 2 females, same data as holotype; 1 female, Hainan, Wuzhishan National Nature Reserve, bush leaves, 17.IX.2019, Zhixin He & Tao Zhang, sweep net, leg.; 1 male, 2 females, Hainan, Ledong, Jianfengling National Nature Reserve, bush leaves, 20.IX.2019, Zhixin He & Tao Zhang, sweep net, leg. (SNNU).

##### Description.

**Male** (Figs 9A, 10A, 11A, C, E, G). Body size small. Head small, slightly broader than anterior margin of pronotum. Frons significantly narrower than antennal scape. Eyes large and protruding laterally. Three apical joints of maxillary palpi distinctly elongate, and fifth joint apically truncated. Pronotum transverse, posterior margin conspicuously broader than anterior one. Tegmina extending over abdominal apex, and six primary veins staggered with many transverse veins between them. Visible part of hindwing is half length of tegmen. Internal tympanum large and long-oval, external one shaped as a small pit. Hind tibia bearing three dorsal spurs on each side.

***Genitalia*** (Fig. 12A–C). Lateral lobes of epiphallus stick-like and apically blunt. In dorsal view, these lobes are straight and hirsute on the inner margin. Ectoparamere bifurcated as two rectangular branches, and the upper larger than lower one. Rami arcuate, very long, and surpass ectophallic and endophallic apodemes.

**Female** (Figs 9B, 10B, 11B, D, F). Resembles male. Body size slightly larger than male. Longitudinal veins of tegmen parallel and forming rectangular cells with some pale transverse veins (filled yellowish brown). Ovipositor falcate and similar to the species described above.

**Coloration.** Body greenish-brown. Head and pronotum brown. Most of the apical three joints of the maxillary pale and proximally darkly color. Tegmen yellowish-brown with green veins. The hind femur bearing an irregular dark pattern.

**Measurements.** Male: BL 5.95–6.26, PL 0.76–0.86, TL 4.74–4.96, HFL 4.26. Female: BL 5.64–6.15, PL 0.78–0.88, TL 4.74–5.20, HFL 4.45–4.88, OL 1.94–2.19.

##### Etymology.

The name refers to the green veins of the species.

##### Distribution

(Fig. 1). China (Hainan, Zhejiang, Yunnan).

##### Remarks.

Specimens of this species were identified as *A.fujianensis* (= *S.fujianensis*) by He et al. (2010), but this is incorrect. The true *A.fujianensis* bears brown veins and a rounded ectoparamere apex, whereas those assumed to be *A.fujianensis* bear green veins and a squared ectoparamere apex. This species is similar with Amusurgus (Amusurgus) xanthoneurus (Chopard, 1940) in having green veins and the pattern of legs and cerci, but differs in the color of the apex of hind femur (black-brown in *A.xanthoneurus* vs yellow-white in *F.viridipennis*) and in the distance between Cu1 and Cu2 of the tegmen in females (extremely narrow in *A.xanthoneurus* vs relatively wide in *F.viridipennis*).

## Supplementary Material

XML Treatment for Flospes

XML Treatment for Flospes denticulatus

XML Treatment for Flospes fujianensis

XML Treatment for Flospes guangxiensis

XML Treatment for Flospes hainanensis

XML Treatment for Flospes viridipennis
